# Visible particles in parenteral drug products: A review of current safety assessment practice

**DOI:** 10.1016/j.crtox.2024.100175

**Published:** 2024-06-09

**Authors:** Frank Liu, Richard Hutchinson

**Affiliations:** a Safe Product Services LLC, Pittsfield, MA, USA; b Janssen Pharmaceuticals, 1400 McKean Road, Spring House, PA, USA

**Keywords:** Particle, Parenteral drug product, Patient safety, Safety assessment

## Abstract

Parenteral drug products (PDPs) are administered extensively to treat various diseases. Product quality plays a critical role in ensuring patient safety and product efficacy. One important quality challenge is the contamination of particles in PDPs. Particle presence in PDPs represents potential safety risk to patients. Differential guidance and practice have been in place for visible (VPs) and subvisible particles (SVPs) in PDPs. For SVPs, the amount limits have been harmonized in multiple Pharmacopeias. The pharmaceutical industry follows the guided limits for regulatory and quality compliance. However, for VPs, no such acceptable limit has been set. This results in not only quality but also safety challenges for manufacturers and drug developers in managing and evaluating VPs. It is important to understand the potential safety risk of VPs so these can be weighed against the benefit of the PDPs. To evaluate their potential risk(s), it is necessary to understand their nature, origin, frequency of their occurrence, safety risk, the risk mitigation measures, and the method to evaluate their safety. The current paper reviews the critical literature on these aspects and provides insight into considerations when performing safety assessment and managing the risk(s) for VPs in PDPs.

## Highlights

• Particles are regarded as contamination representing safety risk to patients.
• There are no regulatory limits to qualify visible particles.
• Particle safety is impacted by various particle factors.
• Particles may cause physical, microbiological, and chemical adverse effects.
• Different approaches may be needed for particle safety assessment.

## 1 Introduction

In the literature, there are different terms for particles. For this review, the meaning of the term particle includes particulate and particulate matter. The presence of particles in parenteral DPs is an ongoing challenge and an intense discussion point within global regulatory agencies and pharmaceutical industry for many years. Even with today’s well controlled manufacturing processes and regulated and well-practiced particle detection technologies through 100 % inspection programs, absolute assurance of the absence of particles can’t be guaranteed with current state of art manufacturing capabilities. The occurrence of particles in parenteral DPs may result from the manufacturing process and manufacturing environment (design, qualification, validation, execution), and post-production handling, storage conditions, transportation, and handling by end users. Also, the nature of the formulations (e.g., therapeutic proteins) may contribute to particle occurrence. Furthermore, as DP container closure systems become more complicated (i.e., drug pumps and autoinjector pens) methodology for detecting VP materials may be more challenging than it was when most parenteral DPs were packaged in simple glass vials.

Particles are contamination as they are not intended and technically unavoidable regardless of dosage forms of DPs (APIC, 2015, PDA, 2017). The particle contamination of DPs has been regarded as an indicator of overall product quality and may impact patient safety and product efficacy. Due to the quality and safety concerns for particles in parenteral DPs, product recall and resulting shortage has happened. Based on US FDA data, particle-related issues led to 22 % of product recalls for parenteral DPs in the period of 2008–2012. In 2014, 16 injectable DPs were recalled as reported by US FDA due to particle contamination (Tawde, 2014). Particle occurrence is also among the most frequent quality reasons to cause DP shortage (Bukofzer et al., 2015, Mazer-Amirshahi et al., 2014). Due to the significant impact, US FDA has permitted the distribution of medications that contain particles found during inspection with the requirement of using a filter prior to administration to eliminate the particle risk to patient (Mazer-Amirshahi et al., 2014).

The harmonized particle definition across USP, EP and JP is that particulate matter in injections and parenteral infusions consists of extraneous mobile undissolved particles, other than gas bubbles, unintentionally present in the solutions (Bukofzer et al., 2015). Based on their source or size, particles can be classified differently. Depending on their source, particles can be classified as inherent, intrinsic, or extrinsic. Inherent particles are those particles that originate from the drug formulation including small particles common to certain high-concentration proteins formulations (e.g., adjuvant material in suspension products, human serum albumin as an excipient, therapeutic proteins, etc.) and other common delivery forms (e.g., emulsions, lipids, etc.). The most representative inherent particles are proteinaceous particles in therapeutic proteins. Intrinsic particles are those particles that arise from sources related to the formulation, packaging, or assembly processes. They are usually inert and unreactive to the DP in nature and expected to have lower risk level because (as per 21CFR 211.65) these materials shall not be reactive, additive, or absorptive (US FDA, 2023b). One example of intrinsic particles is glass particles. Extrinsic particles are defined as those that enter the container or solution during manufacturing, or additive, foreign, unchanging, and not part of the formulation, package, or assembly process. Example extrinsic particles are clothing fragments or skin flakes. They may pose higher risk compared with inherent or intrinsic particles.

Particles are also classified based on their sizes for regulation and risk assessment. They are generally classified into one of two categories: visible and subvisible. VPs are those that can be detected under controlled condition by the naked human eye (USP, 2013). Detection of particles has a probabilistic nature. No single cut-off size can be defined to be visible to the human eye. Many factors can influence the probability of detecting a particle. The detectable size by the human eye ranges from near 0 %, 40 % and 95 % probability of detection to 50, 100, and 200 µm, respectively, under the standard conditions (Mathonet et al., 2016; Perez et al., 2017). As a single-size estimate, the 150-µm threshold has been proposed to be a best-case threshold for human visual identification of particles in injectable DPs (Bukofzer et al., 2015).

Regulatory requirements have been in place globally regarding particle contamination in parenteral DPs. In the United States, the first compendial standard for VPs in drugs was established by the *National Formulary* VI. It stated that injectable solutions were to be “substantially free from precipitate, cloudiness or turbidity, specs or flecks, fibers or cotton hairs, or any undissolved material” (Langille, 2013). When one thinks about this from the perspective of the medical professional administering the parenteral DP, it seems reasonable to expect that the DP does not have VP in it. By extension, if such VP can be seen by the medical professional it seems reasonable to require the manufacturer to implement similar visual inspection processes. When viewed in this light the requirement for being essentially free of VP is more related to quality than safety. The major global regulation on particles has evolved to the current similar requirement. The USP General Chapter <1> stated that “the inspection process shall be designed and qualified to ensure that every lot of parenteral preparations is essentially free from visible particulates” (Mathonet et al., 2016). The EP (Mathonet et al., 2016), in Parenteral Preparations-Injections (0520), stipulates “solutions for injection, examined under suitable conditions of visibility, are clear and practically free from particles.” A similar regulation is seen in JP specifying “injections or vehicles must be clear and free from readily detectable foreign insoluble matters” (Mathonet et al., 2016). These terms can be interpreted interchangeably in literature and indicate implicitly the probabilistic nature of VP identification. For SVPs ranging in size from 10 µm to the visible threshold, the limits are harmonized in the USP, EP, and JP at 6,000 and 600 per container for ≥10 µm and ≥25 µm particles, respectively, in 100 mL containers. For containers larger than 100 mL, limits are 25 per mL and 3 per mL for particles of ≥10 µm and ≥25 µm, respectively (Ishii-Watabe et al., 2015, Perez et al., 2016). For particles <10 µm, no compendial limits would be applicable, presumably this correlates to the approximate size of a human erythrocyte suggesting that particles smaller than an erythrocyte are of less concern because they are unlikely to embolize.

The differential regulatory requirement leads to challenges in managing visible versus SVP from quality and safety perspectives. With the defined limits for SVP in injectable DPs, a clear quantitative target is easily followed to ensure regulatory compliance. Therefore, quality control and compliance satisfy the safety expectation of injectable DPs. However, for VPs, no particle limit has been set by regulatory agencies. The qualitative requirements of “essentially free” (USP), “practically free” (EP), or “free of readily detectable” VPs (JP) represent practical challenge of managing VPs in injectable DPs to ensure regulatory compliance and patient safety.

Patient safety in relation to VPs is an important aspect of parenteral DPs and has been an intensive topic in literature (Barba, 2000, Langille, 2013, Bukofzer et al., 2015, Perez et al., 2016, Perez et al., 2018). Due to the qualitative nature of the regulatory requirements for VPs in parenteral DPs, their safety assessment needs to be performed to ensure patient safety upon administration of parenteral DPs which are in compliance with conditional free requirement of VPs (i.e., “practically”, “essentially”, or “readily detectable”). Additionally, VPs may happen even under the 100 % inspection process (Bukofzer et al., 2015). The current review intends to focus on safety of VPs: how they happen, how to mitigate their risk, what factors impact their safety, what potential safety risk they may pose to patients, and how to evaluate their safety. Two representative types of particles – inert and proteinaceous – are presented to show the complexity of particle safety assessment. It also emphasizes the importance of comprehensive risk assessment and risk management to balance out the DP quality and availability in relation to VPs and benefits that parenteral DPs provide.

## 2 Safety of VPs

There is no published regulatory guidance regarding the potential impact of small numbers of VPs on patient safety. As a result, safety assessment of VPs in DPs has been a self-regulatory and self-assessment practice in pharmaceutical industry. While there has been published literature on anecdotal reports describing exposure to large numbers of particles (Doessegger et al., 2012; Bukofzer et al., 2015) and resulting adverse effects, this literature represents an extreme situation and, as such, do not reflect the potential hazards of more typical particle exposure via commonly administered parenteral drugs.

Three types of well-known particle-related incidents attracted significant attention for particle safety. Total parenteral nutrition (TPN) products provide IV administered nutrition that can be the only source of nutrition for the patient. It has been found that TPN was associated with deaths following sudden and unexplained respiratory distress (Doessegger et al., 2012). One classic example is calcium phosphate precipitated from the nutrient admixtures forming particles. The intravenous (IV) infusion of the TPN is associated with at least two deaths based on the observation of calcium phosphate particles in blood clots in the lungs of the deceased. Another type is IV drug abuse causing pulmonary embolism due to massive injection of VPs. For example, abusers may mis-use solid oral forms of medication which are ground up and injected in the form of a slurry. Talc- and starch-induced pulmonary damages have been reported in drug offenders (Doessegger et al., 2012). The third type is related to intensive care unit (ICU). It is estimated that “patients in ICUs may receive more than a million injected particles > 2 um daily” (Langille, 2013). Given that many ICU patients need continuous infusion of large volumes of parenteral solutions, they are at greater risk of consequences of particle exposure. The commonality across all these particle exposure scenarios is that the large mass of particles was infused into the subjects. These extreme scenarios have questionable meaningful clinical relevance to the more typical scenario of clinical exposure to small amounts of VPs. Significant adverse observations resulting from extreme mass, number, and diversity of foreign particles in drug abuse and critical care situations are special scenarios and these risks are not generally applicable to more typical exposure scenarios.

Particle safety has been extensively studied by employing animal models after extreme exaggerations of clinical exposure, however limited data exists for more clinically relevant exposure scenarios. A significant number of animal studies have been performed to investigate the fate and effects of visible and SVP with different compositions administered intravenously (Barba, 2000). For example, Garvan and Gunner studied particles in injectables by quantifying and classifying particles that were present in intravenous solutions and their physiological effects based on rabbit experiments and human lung tissues taken at autopsy (Garvan and Gunner, 1963, Garvan and Gunner, 1964). Based on the findings in the animal studies, significant attention has been paid to the safety of particles on patients. In these studies, massive quantities of particles were infused. The observations included histologic evidence of injury to pulmonary capillary endothelial cells (Liu et al., 1992), microscopic thrombi in the pulmonary capillaries, microscopic pulmonary granulomata, and hepatic inflammatory effects. Although these studies help understand the pathophysiologic response to IV particle exposures, the significant number of particles administered in these animal studies provides little guidance evaluating the safety risk of commonly encountered small numbers of VPs to patients. Limited data is available on human exposure to and health effects of infused particles in typical medical environments. This makes particle safety assessment challenging given the nature of particles compared with other types of drug impurities (e.g., extractable and leachable substances, residual solvents, elemental impurities).

## 3 Factors impacting safety of VPs

Potential safety risk associated with the exposure of particles are impacted by many factors, including particle characteristics, patient characteristics, and drug posology.

### 3.1 Particle characteristics

Safety of particles is impacted by their characteristics such as size, volume (or dose), surface charge and morphology. Data from human exposure and animal studies has shown that particle size is a critical parameter of potential safety risk following particle exposure. It has effects on particle positioning and distribution in the human body. For IV exposure, particles with size of ≤1 µm have been shown to deposit predominantly in the liver whereas particles with size of 3–6 µm deposit in the spleen and hepatic lymph nodes (Barba, 2000). Particles with size of ≥10 µm pass the pulmonary vascular bed slowly and are expected to be retained in lung unless they can get through the lung to other organs via collateral blood circulation (Barba, 2000). When particles size is ≥50 µm, they are expected to be entrapped in the lung (Barba, 2000). Size dependent biodistribution is related to the blood flow direction and the size of blood vessels. Introduction of particles into the bloodstream through IV administration results in the particles following the flow of blood through the veins, which increase in size in the direction of blood flow, to reach the right side of the heart. From there, particles would be expected to follow the blood flow from the heart to the lungs via the pulmonary artery. The size of arterial vessels within the lung decreases in the direction of blood flow, ending in pulmonary capillaries that have diameters typically in the 6 to 8 µm size range (Langille, 2013). This correlates to the size of a human erythrocyte. As a result, most particles which are larger than this size will become trapped in small pulmonary arterioles or in the pulmonary capillaries while smaller particles can pass through. In some instances, a larger-size particle may pass through the lung via collateral blood circulation. Smaller sized particles that pass through the pulmonary capillaries may travel through the pulmonary vein to the left side of the heart and then through the systemic circulation and deposit in the capillary beds of other organs (e.g., the liver and spleen) where they can be phagocytosed by cells of the reticuloendothelial system (Bukofzer et al., 2015, Alijagic et al., 2022). As the 150 µm and larger particles can be detected by 100 % visual inspection in injectable DPs (Bukofzer et al., 2015), IV administered VPs are expected to be restricted to the lung (Doessegger et al., 2012) causing physical occlusion of blood vessels. Size-related effects have been reported in literature. Introduction of microspheres with size of 40–120 µm was associated with cases of fatal pulmonary complications following therapeutic intra-arterial embolization; however, when the size of the microspheres was increased to 100–300 µm, no fatality was reported over an 8-year study period (Doessegger et al., 2012). Given most organ systems have significant reserve capacity and the human body has clearance mechanisms of clearing larger particles (Langille, 2013), VPs-related clinical consequences are expected to be have low risk unless a large number of VPs are introduced causing significant occlusion of capillaries (i.e., pathological concerns are limited to only those exposures which exceed the threshold for which normal homeostatic mechanisms can compensate).

Particle shape, surface characteristics (charge and area), and hardness also play important roles in particle safety (Xu et al., 2020; Heddagaard and Møller, 2020; Alijagic et al., 2022). Particle shape may affect particle deposition within the human body and its clinical effects. It was proposed that the geometric shape and surface characteristics of the particles impact their lodging capability resulting in different safety risks (Barba, 2000). A long and thin fiber is expected to be more likely to occlude a minor vessel than a spherical particle. Surface characteristics of particle can also impact their fate and effect in the human body. The blood clearance and organ distribution of small particles (1.305 µm) was found to be impacted by their surface charge as shown in an animal study with rats (Wilkins and Myers, 1966). The blood clearance was significant and fast as 99 % of all injected particles were cleared from the blood 15 min after injection. Negatively charged particles stopped in the liver whereas positively charged particles initially accumulated in the lung then stopped in the spleen later. Surface area of particles is another factor to impact their effects. When dogs were exposed to a fixed mass of glass beads, a greater pulmonary dysfunction was observed with smaller particles sizes than larger particle sizes (Niden and Aviado, 1956). A hard particle has a rigid structure whereas a soft particle has a flexible or deformable structure (Langille, 2013). Compared with a soft particle, a hard particle is more likely to cause mechanical occlusion and vascular emboli. The vascular occlusion by a soft particle can be overcome due to its malleable nature which is not owned by a hard particle. As a result, soft particles may cause physiological effects in downstream vital organs.

The chemical composition of a particle can be important in impacting product quality and patient safety. Depending on the type of particles, chemical constituents may leach from the particles and cause adverse effects (Bukofzer et al., 2015, Heddagaard and Møller, 2020, Avery et al., 2023). These leachates are regarded as impurities which may result in direct chemical hazard-related effects and may also indirectly alter the physiochemical properties of DPs via interaction with the active pharmaceutical ingredient or the excipients in product formulation. The nature of the chemical constituents and particle types determines the chemical-related adverse effects. Chemical constituents may leach out from inert materials (e.g., glass and stainless) used for drug manufacturing and packaging. Particles formed from glass and stainless materials are among commonly encountered particles in parenteral formulations (Shearer, 2003; Bukofzer et al., 2015). Oxide of various metal ions such as aluminum, arsenic, barium, and iron are added in glass to serve different purposes for the container closure system. Metal ions could leach from the glass structure. It has been reported that barium can leach from glass vials (Markovic, 2006). Trace amounts of metal contaminants can arise from contact with stainless steel, and the leaching was affected by various factors such as contact surface area, pH, and the presence of metal chelators (Zhou et al., 2011). The safety of chemical leachates from particles needs to be assessed. Different from potential chemical leaching in relation to inert particles, proteinaceous particles represent a unique type of chemical composition. Proteinaceous particles associated with therapeutic protein DPs may cause immunogenicity (Pham and Meng, 2020, Nabhan et al., 2020). The active protein pharmaceutical ingredients in these products have a tendency of forming aggregates from small protein particles. Anti-drug antibodies (ADAs) have been found to be closely correlated with protein aggregates resulting in irreversible drug loss and increased immunogenicity risk. The safety assessment of inert and proteinaceous particles will be thoroughly discussed in Section 5.

### 3.2 Patient characteristics

Patient population and conditions are important factors in relation to particle safety. Patient age plays a key role. Pediatric population has higher particle-related risk than adult population, explained by differential cardiovascular characteristics and therapeutic administration. Neonates or immature preterm infants especially seem to be at risk (Perez et al., 2016). The cardiovascular system undergoes extensive changes until early childhood (Saikia and Mahanta, 2019). Although neonates and adults are same in the pulmonary capillary diameter, density of the capillary bed and diameter of blood vessels are lower in neonates and infants compared with adults. Alveolar numbers increase and reach adult levels at around 8 years of age (Heyman, 1979, Lofthus and Srebnik, 1987). Prolonged parenteral nutrition, and numerous other IV drugs may be required for immature preterm infants to sustain their life and growth. Due to low venous access, multiple drug infusions are administered simultaneously through one single catheter with an extremely low rate of infusion which represents an increased risk of particle formation due to physical or chemical drug incompatibility (Perez et al., 2016, Perez et al., 2018). Additionally, preterm infants are frequently observed to have right to left shunting through the foramen ovale and/or the ductus arteriosus, especially in those with persistent pulmonary hypertension (Storme et al., 2013). In these populations, particles may cause damage to extrapulmonary tissue and the brain.

It has been reported that pre-existing medical conditions may aggravate particle-related effects. Collateral vessels play important roles in reducing particle-caused vascular obstruction (Barba, 2000). It was found that formation of compensatory collateral vessels is reduced in patients with diabetes or on vascular endothelial growth factor inhibitors (Doessegger et al., 2012). In critically ill children, it was found that the infusions of particles may cause alteration to the microcirculation, leading to systemic inflammatory reactions with adverse effects on organs (Boehne et al., 2013). Aberrant anatomies have been reported resulting in abnormal communication between the venous and arterial systems. Two examples are fistulae for renal dialysis or a patent foramen ovale allowing for a right to left shift of blood circulation within the heart (Bukofzer et al., 2015). In such conditions, particles can bypass the lung and its vasculature, and enter directly into the central vascular system potentially cause ischemic stroke and myocardial ischemia and even end organ damage. If the exposure to high amounts of particles occurs through IV route, patients with organ failure are at great risk of significant effects on microcirculation (Lehr et al., 2002). The loss of functional capillary density was significant in the post-ischemic tissue whereas no such loss was observed in normal muscle tissue.

### 3.3 Drug administration

It is well known that the route of drug administration is a critical factor in particle-related safety risk and degree of such risk. IV infusion is a common administration route for injectable DPs. As the DPs are administered into the bloodstream, it can directly introduce particles to vascular system potentially causing direct physical damage, chemical exposure if leaching happens, or infection if the particle is non-sterile. Different from SVP which pass through lungs and diffuse into the different organs (e.g., the liver and spleen), due to their large size (>150 µm), VPs are expected to be trapped in the lung (Barba, 2000). As a result, the major health effects are expected to happen in pulmonary structure and function. Other common routes of parenteral administration, such as intramuscular and subcutaneous, however, represent lower clinical complications unless there is microbial contamination to the particles or high-hazard chemical constituents leaching out. In the case of particle introduction through these routes, the particles would remain in a contained space with slow possibility of migration to different locations. It was reported that subcutaneous exposure to small, inert, and sterile particles would not be expected to cause clinically significant reactions above minor irritation or perhaps a small granuloma (Miliauskas et al., 1993). A similar conclusion was drawn for intramuscular injections based on analysis of 26,294 hospitalized medical patients (Greenblatt and Divoll-Allen, 1978). For other types of less encountered parenteral DPs, such as intraocular or intrathecal, a similar health impact profile is expected though limited data is available. For example, upon the intrathecal administration of drug solutions containing 7–38 particles in size from 15 to 80 µm, the incidence of central nervous system complication was low, and a foreign body reaction may have resulted (Bukofzer et al., 2015).

In most companies, measures have been taken throughout the drug life cycle to reduce particle occurrence and exposure. A significant amount of literature has shown that use of in-line filtration or filter needles is an effective measure of reducing particle exposure and thus their health effects (Jack et al., 2010, Jack et al., 2012, Mathonet et al., 2016). The use of 0.2 µm in-line filters removed most of particles from being injected through intramuscular administration of generic antibiotics (Schaefer et al., 2008). It has been widely reported that in-line filters reduced particle-related health effects such as phlebitis (Doessegger et al., 2012; Bukofzer et al., 2015) and thrombophlebitis (Jack et al., 2010). Importantly, in-line filters showed their effectiveness in reducing complications in neonates such as sepsis, thrombosis, and necrotizing enterocolitis (van Lingen et al., 2007). In a single-center, prospective, and randomized controlled clinical trial with a total 807 critically ill pediatric patients, in-line filters with a size of either 0.2 µm or 1.2 µm averted severe complications such as systemic inflammatory response syndrome and reduced the length of their stay in pediatric intensive care unit and duration of mechanical ventilation (Jack et al., 2012). Similar finding was observed in another clinical study with pediatric cardiac patients showing that 0.2 µm and 1.2 µm in-line filters significantly decreased systemic inflammatory response syndrome, renal and hematologic dysfunction (Sasse et al., 2015). A dynamic particle count system showed that in-line filters significantly removed particles during a pediatric multidrug infusion (Perez et al., 2018). Although filters themselves may shed particles (Falchuk et al., 1985), the benefits of using them in parenteral drug administration are significant. This has been acknowledged in the USP and EP monographs (Mathonet et al., 2016). Additionally, this has been widely practiced as seen in many clinical pharmacies and the drug prescription information if VPs are present in a final container (Doessegger et al., 2012; Bukofzer et al., 2015). However, it may not be applicable in certain scenarios. As novel modalities emerge, for some product types the use of filtration may not be feasible. For instance, cell-based products cannot be filtered because the live cells would be removed. Also, in the case of prefilled drug administration devices (i.e., autoinjectors), there may be limited opportunity to filter the DP after filling into the final container closure system.

### 4.4 Safety risk of VPs

Safety risks of particles associated with injectable DPs have been discussed extensively in literature. A significant amount of medical literature is available investigating particle safety based on *in vitro* and animal studies, human case reports, and clinical studies. As reviewed in the previous section (3.1), particle size is one of the most critical factors impacting health effects of particles. Phagocytosable particles with size range of 0.1–10 µm are believed to be the most biologically reactive in causing cytokine release, a hallmark of inflammatory reaction caused by particles (Liu et al., 2015) yet are not limited by any compendial requirements. Given VPs have large size (>150 µm), they are relatively less reactive than smaller particles, and thus their safety risks are expected to be lower. However, it is still important to evaluate their safety risks to patients. Based on the current knowledge and literature, they may cause 3 types of adverse effects in clinical settings: (1) physical adverse effects, (2) microbial adverse effects, and (3) chemical adverse effects.

### 4.1 Physical adverse effects

Due to their large size compared with SVPs, VPs may lodge and physically occlude the affected vasculature leading to acute, subacute, or chronic reactions which could be localized or systemic. As a localized reaction, injection site reactions have been reported as a common phenomenon as a result of administration of injectable DPs (Thomaidou and Ramot, 2019). While such reactions may be attributable to a variety of etiologies, particles are one of the potential root causes. It is demonstrated as a local reaction appearing as a constellation of symptoms, such as swelling, erythema, pruritus, and pain around the injection site. Most of them are not allergic or immunogenic reactions in nature. They dissipate as treatment is discontinued and are preventable by adjusting the injection techniques, patient education, and training. Injection site reactions have been reported during TPN but haven’t been confirmed by pathological evaluations (Gil and Mateu, 1998). Injection site reactions associated with VPs were mainly reported in animal studies or in relation to drug abuse (Doesseggaer et al., 2012). Injection- or infusion-related local phlebitis is caused by acute inflammation of a vein with symptoms of pain, tenderness, swelling, and redness around the affected area. It has been reported that infusion of large quantities of particles increased the occurrence of chemical phlebitis at the infusion site (Avis et al., 1992).

Mechanical lodging of particles in blood vessels may cause embolism when the particles are larger than the internal diameter of vessels. Pulmonary embolism could happen when large particles obstruct blood flow through the pulmonary capillaries (Bukofzer et al., 2015). The number of blood vessels in the affected organ impacts the degree of damage. If the organ has extensive blood vasculature, the unimpacted vessels may be sufficient to feed the same organ leading to minimal clinical significance. It has been reported that when >30–50 % of the pulmonary vascular bed is occluded by thromboemboli, acute hemodynamic impairment ensues (Doesseggaer et al., 2012). It has been estimated that millions of particles are required to produce clinical signs and symptoms of organ failure or dysfunction (Montagnana et al., 2011). This has been observed in arterial embolization procedures in clinical settings using alcohol embolic agents, collagen-coated acrylic microspheres, and gelatin spheres (Wijeyaratne et al., 2009, Brown, 2004). In these procedure cases, massive, 300–500 µm particles were applied and moved from the arterial injection site into the venous circulation. The intended thromboembolic event happened without significant long-term clinical consequences observed from the administered particles.

A granuloma is a small area of inflammation organized as aggregates of macrophages (Pagán and Ramakrishnan, 2018). It is a protective immunological mechanism against foreign bodies and infectious agents. Particles introduced through drug administration are foreign bodies. The body’s immunological defense mechanism is driven by their size. Particles with size of ≤10 µm are destroyed and can be cleared by phagocytes (Barba, 2000) without formation of granuloma. On the other hand, larger particles than 10 µm are expected to be destroyed by macrophages potentially resulting in formation of granuloma (Pagán and Ramakrishnan, 2018). A well-known example is the age-old medical challenge related to biomedical implants which stimulate foreign body reactions (Avery et al., 2023). Similar to other physiological protective mechanisms, granuloma can also be pathological. There is limited published evidence of clinical events in relation to granuloma caused by VPs from DPs. The particle-related granuloma was mainly reported in drug abusers (as injection site granulomas) and drug addicts (as pulmonary granulomas) (Doessegger et al., 2012; Bukofzer et al., 2015).

These physical safety effects could be caused by VPs. However, a large quantity of the particles would be required to block larger blood vessels or block a large proportion of the vasculature in an organ (Jorens et al., 2009, Montagnana et al., 2011) through IV infusion. Additionally, the lung vasculature has multiple defensive mechanisms to mitigate the adverse effects from VPs (Barba, 2000). Particles are excreted into the sputum and mucus and continuously cleaned from the lung by an active pulmonary mechanism for passing the trapped particles through the capillary walls. The lung has an extensive circulation and ample collateral blood circulation to reduce the effects from particles upon IV exposure.

For other parenteral routes such as subcutaneous, intramuscular, intrathecal, and intraocular administration, VPs would be contained in the limited injection space with no migration. Injection site reaction may occur if there are a significant number of VPs. These adverse effects have been reported mostly in animal studies or in drug abusers. Limited literature is available regarding clinical histopathological events in relation to VPs.

### 4.2 Microbial adverse effects

Infection, febrile response, and inflammation can ensue in the presence of microorganisms or endotoxins. If particles are contaminated with infectious agents, they may lead to localized or systemic infections by serving as a source of bioburden or a nidus for infection. Extrinsic particles represent a greater risk for microbial contamination and endotoxin introduction into the DPs. Lint and fibers from many sources may pick up contaminants that they have been exposed to such as skin scales. Even low levels of bacterial contamination can cause infection. In IV drug abusers, acute infections have been reported upon the massive administration of particles (Passarino et al., 2005).

To eliminate microbial contamination, a good quality system needs to be in place to monitor for potential sources of microorganisms or endotoxin contamination. The sterility assurance of the DPs is critical with manufacturing measures such as terminal sterilization. Additionally, DPs should be routinely tested at final release to meet the sterility requirement as stated in commonly used compendial tests (Bukofzer et al., 2015). However as novel therapeutics evolve, additional CMC challenges may be presented. For instance, cell-based therapeutics cannot be terminally sterilized because the drug is a living cell. With the modern manufacturing process and environment management and robust tests, safety risks associated with microbial contamination or endotoxin introduction have minimal relevance.

### 4.3 Chemical adverse effects

The nature and chemical composition of particles is a very important aspect for particle safety assessment and risk management (Barba, 2000, Bukofzer et al., 2015, Alijagic et al., 2022, Avery et al., 2023). If the constituents in particles leach out, chemical exposure ensues potentially resulting in chemical toxicity depending on the hazard profile of the leachates. Stainless steel particles are commonly observed particles as DP manufacturing equipment is often made of stainless steel. The release of metal constituents from stainless steel cooking utensils and medical appliances has been studied (Santonen et al., 2010). Chromium and nickel could be released from these articles. Specifically, a study has been performed to evaluate metal release from stainless steel 316L particles of various sizes into artificial sweat. It was found that both nickel and chromium were released from the particles. In relation to biologics formulation, the release of iron, chromium, and nickel from stainless steel 316L was impacted by various factors such as pH, buffer species, solution fill volume per unit contact surface area, and metal chelators (Zhou et al., 2011). Glass is used as the primary choice for packaging of parenteral formulations. Oxides of various metal ions (e.g., aluminum, arsenic, barium, iron) are added to glass for specific purposes. Metal ions could leach from glass containers (Pillai et al., 2016).

Leachates from particles formed of stainless steel, glass, and other materials used during manufacturing and for packaging and storage may impact patient safety. They may have direct effects due to their chemical toxicity or indirect effects through interaction with drug formulation (e.g., generating particles or hastening drug degradation). If particles are generated, their safety can be evaluated following the approach mentioned here. If drug degradation occurs, the efficacy of the DPs will be impacted. The safety risk of the leachates needs to be assessed to ensure patient safety.

Toxicological risk assessment (TRA) is an important practice to evaluate the safety of physical or chemical agents upon their exposure. It finds its application in various fields such as pharmaceutical, consumer, food, and medical device industries. It comprises of 4 steps including hazard identification, dose–response assessment, exposure assessment, and risk characterization. Guidance is available from regulatory agencies and international scientific bodies for how to perform TRA (ECHA, 2023, SCCS, 2023, US EPA, 2023). In relation to DPs, TRA is commonly performed to evaluate the safety of impurities such as extractable and leachable substances, residual solvents, and elemental impurities. These process- and product-related impurities may pose safety risk to patients upon exposure. The leachates from particles are leachable substances. There has been published TRA approaches for leachable substances associated with DPs (Broschard et al., 2016, Liu and Hutchinson, 2023, Masuda-Herrera et al., 2023, PQRI, 2021). The challenging aspect for TRA of potential leachates associated with particles is their exposure assessment. Often time, it is unknow how much and what chemical constituents may leach out. The best-case scenario is that a leachable study should be performed to determine the leachable amount. However, as the leachable amount can be also influenced by particle characteristics such as number, surface area, size, etc., it may be challenging to define the accurate exposure amount. One alternative is to apply the worst-case assumption that chemical constituents fully leach out unless it can justify robustly that they are not expected to be released or the release is negligible to cause safety concerns to patient. As seen from Section 5.1, based on the available data of chemical leachates from common particles (e.g., glass, stainless), the leachates may occur at low level which is associated with negligible safety concern. Therefore, the worst-case assumption is needed only in special situations. A qualified toxicologist is required to perform the TRA to ensure patient safety.

Proteinaceous particles represent a unique type of particle when it comes to chemical-related safety risk. Instead of chemical release from the proteinaceous particles, they elicit immune responses themselves as an aggregate entirety. Their safety assessment is covered in Section 5.2.

### 4.5 Safety assessment of representative VPs

Commonly encountered particle contaminants in DPs include fibers, silicone, plastics, rubber, metal particles, glass particles, skin flakes, char particles, lubricant oils, Teflon, graphite, hair, clothing fragments, and paint (Shearer, 2003; Bukofzer et al., 2015). Therapeutic proteins may contain proteinaceous particles (Mathonet et al., 2016). The current paper aims to provide a review on two representative types of particles: inert particles and proteinaceous particles given they are associated with standard manufacturing and container systems (i.e., inert particles) or specific type of DPs (i.e., proteinaceous particles).

### 5.1 Safety of inert particles

#### 5.1.1 Safety of glass particles

Inert particles are typically intrinsic particles as they come from the formulation, packaging, or assembly processes (Bukofzer et al., 2015). Particles of glass and stainless steel are among the commonly encountered inert particles. The materials are not chemically reactive in nature because they typically come from manufacturing contract materials selected to comply with 21CFR 211.65 which specifies that these materials shall not be reactive, additive, or absorptive (US FDA, 2023b). It is well accepted that inert particles pose minimal toxicological risk to patients.

Glass is virtually inert as it is mostly resistant to chemical attack and has thermal resistance. It typically does not react with other substances or break down into the constituents it contains (Gardner and Hahner, 1949). Glass is traditionally used as the primary choice for container of parenteral formulations. Glass ampoules are widely used to package parenteral solutions. Glass particles can form following the opening of glass ampoules (Zabir et al., 2008). As a result, patients may be exposed to glass particles either through injection by subcutaneous, intramuscular, or IV administration routes (Joo et al., 2016, Perez et al., 2016). A great effort has been devoted to strategies to reduce particle contamination while opening glass ampoules (Chiannilkulchai and Kejkornkaew, 2021). For example, the wrapping technique and breaking direction affected the number of glass articles. Both subvisible and VPs were observed.

As reviewed in Section 4, glass particles, if introduced into vascular systems, their exposure may result in localized and systemic health effects such as phlebitis, pulmonary emboli, and granuloma formulation in case of IV administration. For intramuscular and subcutaneous administration, the symptoms may be pain, hematoma formation, acute inflammation and transient nodules (Chiannilkulchai and Kejkornkaew, 2021). These safety effects can be reduced by applying in-line filters or needles (Doessegger et al., 2012; Bukofzer et al., 2015). Patient exposure to a small quantity of VPs is not likely to cause significant injuries (Bukofzer et al., 2015). A specific type of glass particle is glass lamellae or flakes. They are formed from glass delamination process. Their formation is impacted by many factors, such as “…glass vials manufactured by the tubing process at higher heat, drug solutions formulated at high pH, the duration of time the DP is in contact with the glass container, and terminal sterilization processes.” (US FDA, 2023a). Their occurrence in injectable drugs filled in small-volume glass vials has resulted in multiple product recalls in 2010 and 2011. US FDA issued an advisory to drug manufacturers with recommendations to help prevent the formation of glass lamellae. Followed the 2011 Advisory, US FDA analyzed surveillance data from 2008 through 2017 concluding on 03 November 2023 that “FDA’s analysis of available data did not identify any new or increasing safety signals since the advisory was issued in 2011.” It was indicated that the favorable observation resulted from improving glass quality for pharmaceutical packaging.

In a review of metal and non-metal element release (Pillai et al., 2016), various studies have shown that metal (e.g., aluminum, arsenic, and barium) and non-metal (e.g. silicon) were released from glass containers. For example, aluminum and arsenic were released when glass containers were subjected to 210 °C for 30 min. The release of these elements happened either in exaggerated conditions or resulted in residual levels of elemental release. There are regulatory limits for the residual levels of critical elements that the glass manufacturing industry is required to comply with. For example, US FDA stipulates that the limit for aluminum and arsenic is 25 µg/L for small volume parenteral administration and 0.1 mg/L for IV administration, respectively (Pillai et al., 2016). Therefore, chemical constituents, if released from glass particles under biological conditions, are not expected to be clinically significant.

##### 5.5.1.2 Safety of stainless particles

Introduction of metal particles into DPs happens mainly during the manufacturing process. As most drug manufacturing equipment is constructed of stainless steel, stainless-steel particles are the main type of metals particles if detected in DPs. Stainless steels are a complex group of iron-based alloys containing at least 10.5 % chromium (could be up to 20 %) and a maximum of 1.2 % carbon. Another major alloying element is nickel with the maximum level up to 38 %. It also contains other elements including carbon, sulfur, aluminum, molybdenum, tungsten, nitrogen, copper, titanium, niobium, zirconium, cerium, manganese, calcium and silicon (Santonen et al., 2010).

Metal elements, if leached out, may have a direct safety impact to patients due to their chemical hazards and indirectly affect efficacy of DPs via altering their physicochemical properties. Their direct effects result from their chemical hazard. At least two types of indirect effects may result. They can interact with formulation ingredients to form unfavorable identity such as particles (Markovic, 2006, Bee et al., 2009; Langille, 2013). Metal ions may also interact with protein through electrostatic interactions or covalent binding resulting in degradation of therapeutic protein (Passerini et al., 2006). Therefore, their safety needs to be assessed if they leach out of stainless particles.

Among the different grades of stainless steels, the grade of 316L is commonly used in biopharmaceutical applications (Zhou et al., 2011). It is an alloy mainly composed of iron, nickel, and chromium with minor amounts of manganese and molybdenum. Metal release from 316L stainless steel particles have been studied. In one study, the metal release from stainless steel 316L particles with various sizes (up to 44 um) into artificial biological media such as sweat was investigated. It was found that the total metal release into different media is generally very low in weight after one-week incubation (0.065 % in artificial lysosomal fluid (pH 4.5), <0.004 % in PBS, <0.008 % in sweat). Iron was found to be the dominant released element with a 10-fold higher release rate than chromium and nickel (Midander et al., 2006). In another study with stainless steel 316L particles (<45 µm and <4 µm), the nickel release level from <45 µm and <4 µm particles was <0.007 µg/cm^2^/week and <0.01 µg/cm^2^/week, respectively, after 168 h exposure in artificial sweat (pH 6.5). When the test was performed in artificial tear fluid (pH 8.0), the nickel release rates after 24 and 168 h of exposure were <0.007 µg/cm^2^/week regardless of the particle sizes. For iron, the average release rate after 168 h exposure in artificial sweat was 0.1 µg/cm^2^/week. Other metals were also released but the amounts released were very low (Midander et al., 2007a).

A comprehensive review has been performed on metal release from stainless steel (Santonen et al., 2010). The concerned stainless-steel made articles include stainless steel cooking utensils, stainless steel medical appliances (e.g., orthodontic appliances and prosthetic implants), stainless steel items that have contact with skin (e.g., door handles, bottle openers, key rings), stainless steel in occupational inhalation scenarios for lung and gastric exposure, and stainless-steel particles. From these studies, iron, chromium, and nickel are the main metal elements for release. In general, the release level was very low, representing low risk. The studies with different grades of stainless steels showed that the differences in chromium and nickel release rates were very small. Additionally, it seems that different surface finishes caused only small differences on the release rates.

Based on available data, it was indicated that the health hazard associated with very low metal release from 316L stainless steel particles is low (Santonen et al., 2010). Given the similarity of metal release from different grades of stainless steels, the metal release data from 316L stainless steel particles can be leveraged to assess the safety of different grades of stainless-steel particles when assessing safety particles composed of other grades of stainless steel. The permitted daily exposures (PDEs) have been defined for metal elements as contaminants in DPs (ICH, 2022). If desired, a quantitative safety assessment can be performed based on the exposure information from the stainless-steel particles (Midander et al., 2006, Midander et al., 2007b) against the ICH defined PDEs, or other toxicological limits. However, a general observation from literature report is that the metal release level is very low, and thus safety risk associated with metal release from stainless stain particles is minimal.

For the indirect effects (i.e., particle formation or binding to protein resulting in degradation of therapeutic protein), released metal ions may affect safety and efficacy of DPs. These aspects should be built into quality control and management program to prevent it from happening. If it happens, the resulting consequences (i.e., particles or degraded therapeutic protein) need to be evaluated for their safety.

### 5.2 Proteinaceous particles

Proteinaceous particles may be present in therapeutic proteins at release or form during storage. Therapeutic proteins exhibit a propensity for self-association resulting in the formation of aggregates with size range from nanometers to microns. This is an inherent molecular property that cannot be fully overcome. An additional contributing factor is that high dose of therapeutic proteins (i.e., tens to hundreds of milligrams protein per dose) are often administered to reach efficacy enhancing molecular interactions and thus proteinaceous particle formation. Packaging components can also be a contributor. For example, silicone oil in pre-filled glass syringes can stimulate protein aggregation to form proteinaceous particles (Gerhardt et al., 2014). Regulation has recognized that fully eliminating proteinaceous particles from therapeutic proteins at release or during storage is a challenge. Given the high benefit/risk ratio of protein therapies to patients, EP has revised the requirement for DP appearance for monoclonal antibodies from “without particles” to “without VPs unless otherwise authorized or justified” (Mathonet et al., 2016).

Protein aggregation occurs as a result of interaction between monomeric proteins during processing, shipping, storage, and administration of therapeutic proteins (Siddiqi et al., 2017). It is a complication which is composed of multiple steps (Roberts, 2014) and may involve different mechanisms (Philo and Arakawa, 2009). It has been an important topic in literature that protein aggregates may cause immune responses such as hypersensitivity and anaphylaxis (Roberts, 2014). Both the innate and the adaptive immune system elicit or enhance immune response to protein aggregates similar to pathogens (Joubert et al., 2011). The innate immune system recognizes repeating patterns of molecular structure by means of receptors that bind to features of these regular patterns. Like pathogens, protein aggregates contain repetitive molecular patterns. Additionally, they might also interact with components of the complement system and Fe gamma receptors and in mediating the innate response. The adaptive immune system can recognize the epitope and additional features like affinity contained in protein aggregates forming anti-drug antibodies (ADAs) through T-cell dependent or independent manner (Rosenberg, 2006; Ratanji et al., 2014). ADA has been a challenge for the development of therapeutic proteins. They may induce hypersensitivity reactions, cytokine release, or other acute reactions regardless of their neutralizing capability. Protein aggregate also reduces efficacy of therapeutic proteins due to less protein drug substance available as a result of protein aggregation and ADA-associated neutralizing activity. ADA also may change the pharmacokinetic and pharmacodynamic behavior of the therapeutic proteins.

Protein aggregate-induced immune response is impacted by various factors. Protein aggregates with high molecular weight arrays of antigen can efficiently elicit an antibody response independent of T helper cell whereas less ordered protein aggregates require T helper cell (Dintzis et al., 1989, Moussa et al., 2016). Multimerization is a prerequisite for protein aggregates to induce immune responses. Larger multimers with moleuclar weight >100 kD and >20 ligands per aggregate are more efficient than low molecular weight aggregates in inducing immune responses (Rosenberg, 2006). Additional factors include the presence of neoepitopes, differences in glycosylation, and existence of contaminants with immunomodulatory activity. In general, therapeutic versions of endogenous proteins are less immunogenic as the immune system has been tolerized to them. Additional factors such as drug treatment regimen (e.g., dose, route, and frequency of administration) and the disease state also are critical factors in relation to protein aggregates associated immune responses in patients. Knowing characteristics of protein aggregates is important in evaluating their potential for immunogenicity.

Immunogenicity associated with protein aggregates has been extensively studied using *in vitro* and animal models. Different types of cell models were used to evaluate protein aggregate-related immunogenicity (Jawa et al., 2013). Among them, peripheral blood mononuclear cells (PMBC) were widely used. The *in vitro* studies with PMBC-based systems showed immune response associated with protein aggregates. Different transgenic animal models find their significant use in investigating immunogenicity of human proteins and their aggregates. It was found in these transgenic models that ability of protein aggregates to break immunologic tolerance is impacted by various factors including the nature of the protein molecule, the characteristics of protein aggregates, the extent and type of chemical modification, the administration route, and the animal species used (Moussa et al., 2016).

Studies conducted both *in vitro* and in animals provided insightful information to facilitate our understanding of immunogenicity potential by protein aggregates. However, the translatability of the immunogenicity observed in the *in vitro* and *in vivo* studies to human remains debatable. The *in vitro* models lack many of the components that are important or may contribute to the immunogenicity *in vivo* such as the presence of other cell types with biologically relevant ratios, exposure to surrounding tissues, and the influence of biodistribution and circulation. Clinical relevance of animal models has been questioned for their poor predictivity of immunogenic responses in humans because of differences in animal and human immune systems, the higher potential for immune-competent animals to recognize human proteins as foreign antigen, and greater frequency and severity of the immunogenic response to a human or humanized protein in animal models (Bugelski and Treacy, 2004, Leach et al., 2014, Kronenberg et al., 2017). It was found that wildtype and transgenic animals showed different immune responses to protein aggregates (Fradkin et al., 2009). Moreover, a significant number of protein aggregates generated under artificially stressed conditions were applied with concentrations of orders of magnitude greater than those found in typical dose of therapeutic proteins. Due to their lack of being representative of actual manufacturing, commercial processing, and administration of therapeutic proteins for protein aggregate occurrence, the immunogenicity finding observed in these non-clinical studies may not be clinically meaningful.

Due to lack of validated *in vitro* and in animal models, the immune response potential of protein aggregates in therapeutic proteins need to be evaluated ultimately in clinical studies. Additionally, it is critical in clinical studies that the properties of protein aggregates are well characterized and understood to establish acceptable profiles (i.e., level and attributes) of protein aggregates for patient exposure and safety assessment. Also, pharmacovigilance data can provide insight on potential association between protein aggregates and immunogenicity. The association between protein aggregates and immunogenicity by therapeutic proteins has been recognized (Rosenberg, 2006, Ratanji et al., 2014). However, such findings were observed for early-era therapeutic proteins such as, human gamma globulin (hGG) (Ellis and Henney, 1969), human growth hormone (hGH) (Moore and Leppert, 1980) interferon alpha (IFNα) (Ryff, 1997), and Factor VIII (FVIII) (Jacquemin and Saint-Remy, 1998). As various risk factors influence the immunogenicity observed in these cases, the observation could be correlative but not causative (Moussa et al., 2016). However, these findings suggested that protein aggregates may have impacted the immunogenicity observed in the clinical settings. The precise underlying immunological and biochemical mechanisms responsible for the impact is not fully understood (Ratanji et al., 2014).

Safety assessment of immunogenicity by protein aggregation in therapeutic proteins is challenging and complicated as the immunogenicity could be impacted by many factors associated with protein particles (i.e., size, amount, morphology, chemical modification), protein composition and structure, products, patients, and treatment regimen. When performing safety assessment of protein aggregates, all these factors need to be taken into consideration. However, significant evidence has shown that protein aggregates associated with therapeutic proteins is not expected to be a significant clinical concern. A small number of proteinaceous particles is not expected to lead to adverse effects on product safety. This has been observed by a review of monoclonal antibody products approved in the EU and US (Mathonet et al., 2016). Probably, one potential reason is related to more modern refined manufacturing techniques and stringent quality control programs that limit the level of proteinaceous particles in therapeutic proteins. In contrast to some early-era therapeutic proteins, there is little to no clinical evidence available in relation to the adverse immune effects by protein aggregates (Moussa et al., 2016). Additional programs including strict scrutiny of DPs before administration and pharmacovigilance help reduce the chance of patient exposure to proteinaceous particles. Proteinaceous particles are extremely difficult to be fully eliminated from therapeutic proteins. Although all available tools and measures can be used to reduce their risks to patients, a greater amount of research to help understand the underlying mechanism remains critical. Additional improvements and refinements of quality and stability programs should be sought for therapeutic proteins to manage the occurrence of proteinaceous particles.

## 6 Conclusion

Particle contamination of DPs has long been an important topic for quality, efficacy, and safety reasons. The first line of defense should be devoted to minimizing their occurrence during manufacturing, shipping, storage, and handling to meet the regulatory requirements as stipulated quantitatively for SVPs (i.e., size-based limit) and qualitatively for VPs (i.e., “essentially free”, “practically free”, or “free of readily detectable”) in USP, EP, and JP. Although stringent quality control measures and refined modern manufacturing techniques have been in place, it is challenging to fully eliminate their occurrence in DPs. Per the ICH Q9 (2023), risk needs to be assessed and managed for quality attributes of DPs. In relation to particles, the risk assessment is composed of two modules: the probability of occurrence of harm and the severity of that harm. Different from SVPs for which quantitative regulatory limits have been defined, the regulatory requirement on VPs is qualitative in nature resulting a significant challenge for their risk assessment.

Many factors impact VP safety including characteristics of particles and patients and drug administration. The safety concerns of VPs are mainly related to parenteral administration such as IV, subcutaneous, intramuscular, intrathecal, and intraocular. If visible particle exposure happens, they may cause physical, microbial, and chemical adverse effects. Particle safety assessment should be performed thoroughly in all these aspects to ensure patient safety. The types and nature of VPs are critical aspects for their health effects. Particles from contact surfaces (e.g. glass and stainless particles) of primary containers are among the most commonly encountered ones. As they are not expected to be reactive chemically, and since sterility requirement for DPs likely preclude bacteriological concerns, the most significant potential safety concerns is mechanical occlusion of vascular system if a significant number of VPs is administered. Proteinaceous particles represent a unique type of safety risk-immunogenicity. They are formed when protein aggregates form in therapeutic proteins. They may elicit immunogenicity by interacting with innate and adaptive immune system. The generation of ADAs is not a rare phenomenon upon administration of therapeutic proteins which are not endogenous origin. Proteinaceous particles may impact efficacy, safety, and quality of therapeutic proteins as a result of less available protein drug substances, change of the pharmacokinetic and pharmacodynamic behavior of the drug, and ADA formation. Various factors such as characterization of proteinaceous particles, overall immunogenicity adverse events relative to millions of doses administered need to be taken into account in evaluating their safety potential.

Although a significant amount of *in vitro* and animal studies has been performed to evaluate the safety of various sizes of particles including VPs, the clinical relevance of the results is questionable due to experimental deficiencies. A large number of particles were typically administered in these studies and thus the studies do not represent the actual particle exposure associated with typical parenteral drug administration where patients have low chance of particle exposure. For proteinaceous particles, their translatability of immunogenicity from *in vitro* and *in vivo* to human is debatable. Cellular models are not capable of fully reflecting the physiological systems which are needed for immune response. The difference in animal and human immune systems makes it difficult to predict immunogenicity in humans using animal models.

Particle-related health effects have been reported mainly in several well-known situations: TPN, drug abuse, and ICU patients. Other than these situations, there have been limited literature records of clinical effects associated with routine administration of parenteral DPs containing particles. The true patient risk in relation to parenteral drug administration is limited at the current profile of particles contained therein. This supports the hypothesis that a small number of particles, proteinaceous or not, has overall low risk to patients. On the other hand, parenteral DPs generally deliver great benefits to patients. With continued advances in manufacturing techniques, more stringent quality control, increased particle monitoring prior to administration by health professions, implementation of in-line filtration for drug administration, and continued pharmacovigilance program, particle contamination and exposure has been become rarer, but not been eliminated. Given that products comply with current state of art manufacturing expectations, and compendial requirements, the clinical effects in relation to VPs in parenteral products are expected not to be significant. To assess and manage VPs, in a parenteral DP, acceptable levels of VP need to first comply with state-of-the-art GMP principles and compendial requirements, second be minimized to the extent practical and lastly be justified on a case-by-case basis taking into account the potential risk(s) in comparison to the benefit of the DP.

## CRediT authorship contribution statement

**Frank Liu:** Conceptualization, Writing – original draft. **Richard Hutchinson:** Writing – review & editing.

## Declaration of competing interest

The authors declare that they have no known competing financial interests or personal relationships that could have appeared to influence the work reported in this paper.

## Data Availability

No data was used for the research described in the article.
